# Non-Invasive Separation of Alcoholic and Non-Alcoholic Liver Disease with Predictive Modeling

**DOI:** 10.1371/journal.pone.0101444

**Published:** 2014-07-02

**Authors:** Jan-Peter Sowa, Özgür Atmaca, Alisan Kahraman, Martin Schlattjan, Marion Lindner, Svenja Sydor, Norbert Scherbaum, Karoline Lackner, Guido Gerken, Dominik Heider, Gavin E. Arteel, Yesim Erim, Ali Canbay

**Affiliations:** 1 Department of Gastroenterology and Hepatology, University Hospital, University Duisburg-Essen, Essen, Germany; 2 Faculty of Medicine, Hacettepe University, Ankara, Turkey; 3 LVR-Clinic, University Hospital, University Duisburg-Essen, Essen, Germany; 4 Institute for Pathology, Medical University Graz, Graz, Austria; 5 Department of Bioinformatics, Straubing Center of Science, Straubing, Germany; 6 Department of Pharmacology and Toxicology, University of Louisville Health Sciences Center, Louisville, Kentucky, United States of America; 7 Psychosomatic and Psychotherapeutic Department, University Hospital Erlangen, Erlangen, Germany; Institute of Medical Research A Lanari-IDIM, University of Buenos Aires-National Council of Scientific and Technological Research (CONICET), Argentina

## Abstract

**Background & Objective:**

Currently, a major clinical challenge is to distinguish between chronic liver disease caused by metabolic syndrome (non-alcoholic fatty liver disease, NAFLD) from that caused by long term or excessive alcohol consumption (ALD). The etiology of severe liver disease affects treatment options and priorities for liver transplantation and organ allocation. Thus we compared physiologically similar NAFLD and ALD patients to detect biochemical differences for improved separation of these mechanistically overlapping etiologies.

**Methods:**

In a cohort of 31 NAFLD patients with BMI below 30 and a cohort of ALD patient with (ALDC n = 51) or without cirrhosis (ALDNC n = 51) serum transaminases, cell death markers and (adipo-)cytokines were assessed. Groups were compared with One-way ANOVA and Tukey's correction. Predictive models were built by machine learning techniques.

**Results:**

NAFLD, ALDNC or ALDC patients did not differ in demographic parameters. The ratio of alanine aminotransferase/aspartate aminotransferase - common serum parameters for liver damage - was significantly higher in the NAFLD group compared to both ALD groups (each *p*<0.0001). Adiponectin and tumor necrosis factor(TNF)-alpha were significantly lower in NAFLD than in ALDNC (*p*<0.05) or ALDC patients (*p*<0.0001). Significantly higher serum concentrations of cell death markers, hyaluronic acid, adiponectin, and TNF-alpha (each *p*<0.0001) were found in ALDC compared to ALDNC. Using machine learning techniques we were able to discern NAFLD and ALDNC (up to an AUC of 0.9118±0.0056) or ALDC and ALDNC (up to an AUC of 0.9846±0.0018), respectively.

**Conclusions:**

Machine learning techniques relying on ALT/AST ratio, adipokines and cytokines distinguish NAFLD and ALD. In addition, severity of ALD may be non-invasively diagnosed *via* serum cytokine concentrations.

## Introduction

One of the major clinical challenges currently is to distinguish chronic liver disease on the basis of obesity from liver damage derived from long term or excess alcohol consumption. Both entities comprise a metabolic injury to the liver either as non-alcoholic fatty liver disease (NAFLD) or as alcoholic liver disease (ALD). Both diseases initially present as steatosis [1], [2], but can progress to steatohepatitis, fibrosis and subsequently cirrhosis, the latter greatly increases the risk of hepatocellular carcinoma. The histologic changes caused by these diseases are similar enough that without close inspection of general physiology, co-morbidities and patient history, it is often difficult to determine the major damaging component in each individual case.

Delineating the cause of fatty liver disease has a critical impact on patient care. There is no universally accepted mechanism-based therapy to halt or reverse either ALD or NAFLD, and primary therapies focus on lifestyle modifications to reduce the proximate cause of the diseases. For example, reduction or cessation of alcohol consumption is an effective hallmark of therapy for ALD, regardless of stage [3]. However, despite many psychiatric methods to support patients willing to improve their health *via* lifestyle changes, relapses are very common. Similar limitations exist for treating NAFLD, apart from bariatric surgery. Indeed, although lifestyle changes to reduce BMI is also an effective therapy for NAFLD, this approach is confounded by difficulties in achieving effective and permanent weight loss. Furthermore, NAFLD is not recognized by all providers of medical care or in administrative boards for treatment guidelines. Due to the increasing incidence and expected further rise as predicted by increasing adolescent obesity in industrialized societies, the latter topic is of particular importance [4].

The difficulty in distinguishing between ALD and NAFLD also impacts therapies for end-stage liver disease, namely liver transplantation. Barring biopsy, it is difficult to stage the liver disease, which is critical for prioritizing care. Furthermore, many transplantation guidelines require at least 6 months abstinence from alcohol for a patient with chronic ALD to be eligible for liver transplantation. Indeed, willingness to cease consumption of alcohol is an obligatory statement for ALD patients to be at least listed for a transplant. Although less rigorous rules are generally applied to NAFLD patients, it may still be difficult to prove true NAFLD (i.e., absent alcohol consumption, especially since physical and metabolic comorbidities of NAFLD and ALD often overlap (i.e. overweight, diabetes) [5]. In the end, many NAFLD patients face the potentially incorrect diagnosis of ALD from primary health providers. This potential misdiagnosis could therefore prohibit the option of liver transplants for NAFLD-derived end-stage liver disease. The above described situation is further complicated by conflicting results indicating moderate alcohol consumption may either ameliorate or aggravate underlying NAFLD [6], [7]. These issues emphasize a critical need to develop a clear and reliable clinical assay to separate predominantly non-alcoholic *vs.* alcoholic fatty liver damage.

The rate of alcohol metabolism is too rapid to use as an index, barring active inebriation at the time of presentation. Although psychiatric assessment of a patients' alcohol consumption may be a feasible option, it relies in part on self-reporting, in a patient cohort infamous for concealing or minimizing their addiction [8].

Machine learning refers to a variety of techniques dealing with pattern recognition based on models for classification and prediction of novel unseen data. Machine learning incorporates the automatic construction of models and application of these models to new data and hence is closely related to the field of data mining. Statistical methods and machine learning techniques have been widely used in biomedical research to evaluate and analyze data. In principle, machine learning techniques are based on data given as a set of attributes, which are assigned to a specific predefined class (i.e. non-alcoholic or alcoholic liver disease, as in the present study). A classification model generated by machine learning describes the mapping from a set of attributes to the corresponding class. Once generated, this model can be used to predict new unseen data, thus enabling classification relying on a set of attributes. Among other considerations this would be an initial step towards personalized therapy for a given patient. A major advantage above other statistical methods is that machine learning techniques provide a robust multivariate approach with multiple features taken into account simultaneously, without the need for variable selection.

In the present study, the focus was on discerning NAFLD and ALD patients with similar physiological and metabolic features in cohorts of patients with similar BMIs. An added goal was to attempt to distinguish between cirrhotic and non-cirrhotic ALD by serum derived variables. These variables allow quick retrieval in a clinical setting and give clear objective measurements for disease assessment. Four different machine learning techniques were applied to analyze predictive possibilities of the collected non-invasive parameters.

## Material and Methods

### Patients

The study protocol conformed to the revised Declaration of Helsinki (Edinburgh, 2000), was approved by the local Institutional Review Board (Ethik Kommission am Universitätsklinikum Essen; file number 09–4252), and all patients gave written informed consent to study participation prior enrollment.

NAFLD patients were enrolled in the hepatologic outpatient clinic at the University Hospital Essen from 2009–2013. Enrollment criteria were a sonographically present steatosis and absence of any known or detected chronic or acute liver disease (viral, autoimmune, toxicity). Exclusion criteria were a BMI above 30, self reported alcohol consumption above 20 g/day for women or 40 g/day for men, or an age below 18years.

ALD patients were enrolled in the LVR-Clinic at the University Hospital Essen and in the addiction therapy unit of the Fliedner Clinic, Düsseldorf. Patients were recruited during the assessment for liver transplantation [8] or during inpatient rehabilitation for chronic alcohol abuse, respectively. Enrollment criteria were a proven history of alcohol consumption. Individuals aged <18 years, patients with a history of organ transplantation, a history of malignancy within the previous five years, drug abuse within the previous year, autoimmunity, genetic disorders, and therapies with immunosuppressive and/or cytotoxic agents were excluded. ALD patients were grouped according to ultrasonographically detectable cirrhosis into patients without (ALDNC) or with (ALDC) cirrhosis.

All enrolled patients were examined physically and ultrasonographically, and a complete set of laboratory parameters was obtained *via* the Central Laboratory Unit of the University Hospital Essen or the Fliedner Clinic. Transient elastography of the liver was measured with a FibroScan system.

### Biochemical assays and ELISAs

Concentrations of serum M30 (for apoptotic cell death) or M65 (overall cell death) were detected with M30 Apoptosense ELISA or Epideath ELISA (Tecomedical group, Switzerland), respectively, according to the manufacturers' instructions. Serum concentrations of hyaluronic acid, adiponectin, and TNF-alpha were assessed with Hyaluronic Acid Test Kit (Corgenix, Bloomfield, CO, USA), the Human Adiponectin/Acrp30 Quantikine ELISA Kit, and Human TNF-alpha Quantikine ELISA Kit (both R&D, Minneapolis, MN) respectively, according to manufacturers' instructions.

### Statistics

All data are expressed as means ± SEM unless specified otherwise. Graphical display gives all single data points as dot plot including mean and SEM. Statistical significance (*p*<0.05) was assessed by One-way ANOVA with Tukey's correction for multiple comparisons. All statistical analyses were performed using GraphPad Prism (Version 5.00, GraphPad Software, San Diego, CA, USA).

### Machine learning

Four different machine learning techniques were employed for evaluation of prognostic properties of available parameters: logistic regression, decision trees (DT), support-vector machines (SVM) and random forests (RF). Mean imputation was performed to compensate for missing values. The SVM is probably one of the most widely used machine learning methods. In their basic form (using the implementation in the R package *kernlab* with the *vanilla kernel*, i.e. the identity function), SVMs are based on the concept of linear separation of data. Thus, they are similar to other linear classifiers, such as the logistic regression. However, SVMs also try to maximize the margin between the two classes [9]. In contrast to the other models, RFs [10] are classifier ensembles, i.e. they are built out of a set of decision trees. For calculation of the RFs the implementation in the *randomForest* package of R (www.r-project.org) was used. Each RF consisted of 2000 randomly and independently grown DTs. When using the trained RF for prediction, an unseen instance was assigned to the positive class voted for by at least 50% of the trees. In addition to a high prediction performance, RFs are able to estimate the importance of features. The importance of each variable for the correct classification was assessed by determining the decrease in Gini impurity [11]. Single DTs were evaluated using the R package *rpart*. The logistic regression model was built in R as well.

All models were validated using ten-fold leave-one-out cross-validation [11] to assess the mean prediction sensitivity, specificity, and accuracy (see formulas below) and the ability to generalize to unseen instances.

For each test in the cross-validation, the sensitivity (SN), specificity (SP), and accuracy (AC) were calculated according to:
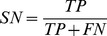


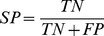


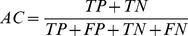
with true positives TP, false positives FP, false negatives FN and true negatives TN. Receiver Operating Characteristics (ROC) curves [12] and the corresponding area under the curve (AUC) with ROCR [13] were calculated (for SVMs, logistic regression and RFs). The ROC curve was built by plotting sensitivity vs. specificity for every possible cut-off between the two classes. For the DTs accuracy was calculated instead of the AUC.

The models were tested for significance using a permutation test. The AUC distribution (for the DTs accuracy was used) for each classifier was calculated by ten-fold leave-one-out cross-validation. 1000 ( = N) random permutations of the class labels were generated and the classifiers were trained and evaluated again. Each of the resulting AUC distributions of the permutation was compared with the real AUC distribution using Wilcoxon Signed-Rank test. The number k of permutations for which the mean AUC had no significant differences compared to the real AUC was counted for each classifier. The p-value of the permutation test was calculated by 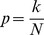



The null hypothesis was that there are no differences between the compared classifiers.

## Results

### Alcoholic and non-alcoholic liver disease can occur on a similar basis of patient demography

NAFLD patients were selected on the basis of a BMI below 30. Patients with alcoholic liver disease were distributed according to presence of sonographically verified cirrhosis (ALD non-cirrhotic  =  ALDNC; AFD cirrhotic  =  ALDC). Distributions of gender, age and incidence of diabetes for patients are given in **table 1**. Due to the selection of NAFLD patients, there were no statistically significant differences in BMI between the patient groups. Although gender distribution tended slightly towards a higher proportion of females in the ALDNC group, the difference did not reach significance.

**Table 1:** Demographic and basic health data of the investigated study groups.

	NAFLD^1^	ALDNC^2^	ALDC^3^
N	31	51	51
Gender ratio	f: m = 15: 16	f: m = 16: 35#	f: m = 24: 27
Age (yrs.)	45.8±2.7###	49.2±1.2#	54.9±1.1
BMI (kg/m^2^)	25.6±0.6	25.3±0.6	25.3±0.9
Incidence of diabetes	2 (9.5%) n = 21	2 (3.9%)##	7 (13.7%)

1 non-alcoholic liver disease;

2 alcoholic liver disease without cirrhosis;

3 alcoholic liver disease with cirrhosis;

# *p*<0.05 *vs.* ALDC;

## *p*<0.01 *vs.* ALDC;

### *p*<0.001 *vs.* ALDC.

### Clinical liver parameters allow discrimination between NAFLD and ALD

**Figure 1:**
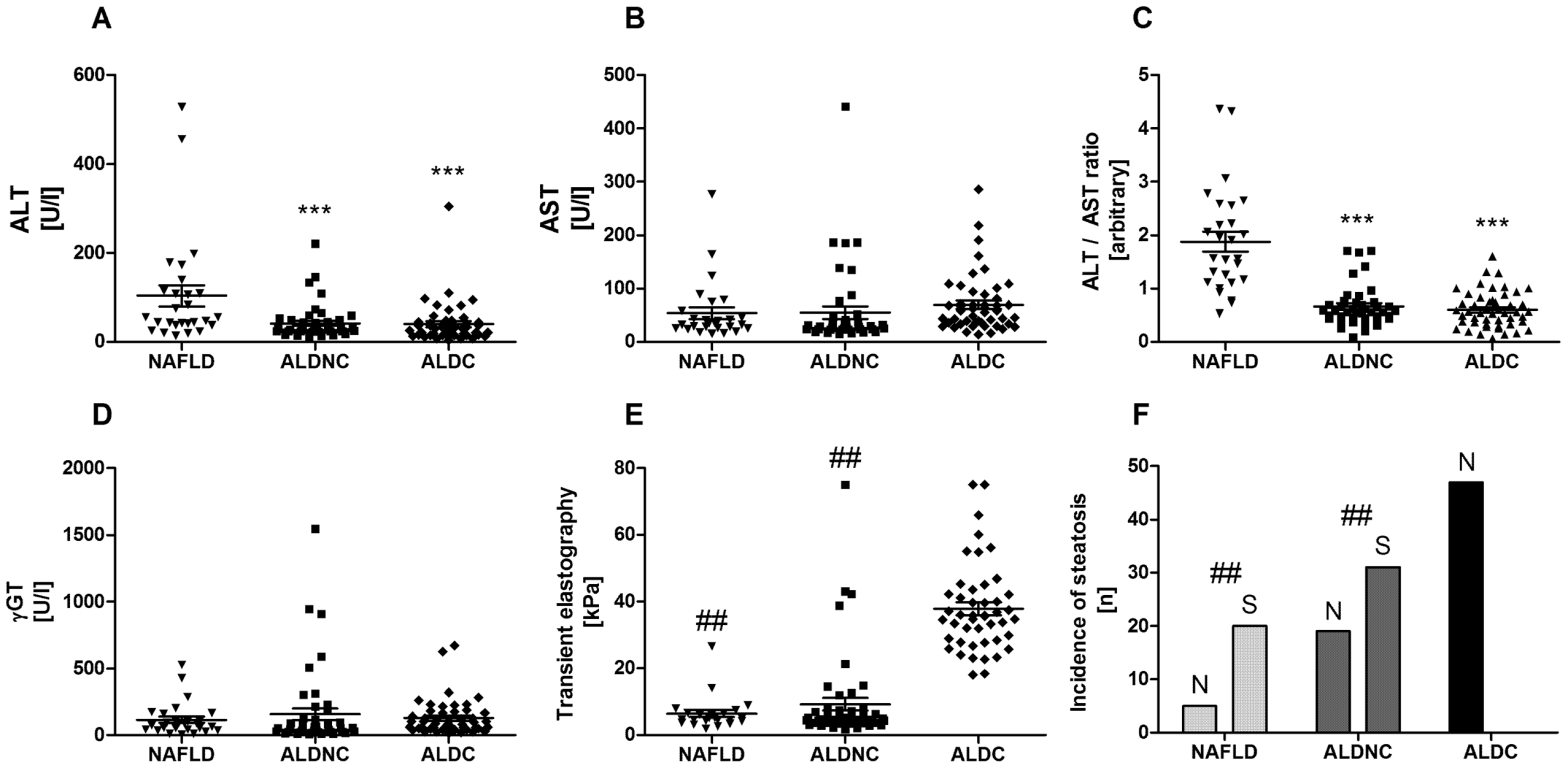
General liver damage parameters do not differ between NAFLD and ALD. Classic serum parameters of liver damage, transient elastography, and sonographically diagnosed steatosis were assessed in NAFLD patients and ALD patients with (ALDC) or without (ALDNC) cirrhosis. While AST (B) and γGT (D) did not differ between the groups, ALT (A) and especially the ALT/AST ratio (C) were significantly higher in NAFLD patients than in both ALD groups. In contrast transient elastography (E), as measure for fibrotic/cirrhotic alterations, and incidence of steatosis (F) were similar in NAFLD and ALDNC patients. ALDC patients exhibited significantly higher transient elastography values and lower incidence of steatosis. *** = *p*<0.0001 *vs.* NAFLD. ## = *p*<0.01 *vs.* ALDC.

Standard serum parameters of liver damage were collected for all patients. As previously described [14], ALT was significantly higher in NAFLD patients compared to ALD, regardless of their cirrhotic status (**Fig. 1A**). AST and γGT did not differ significantly between the groups (**Fig. 1B,D**). The ratio of ALT to AST allowed a very clear discrimination of the NAFLD cohort from ALD patients (**Fig. 1C**). Both non-cirrhotic groups (NAFLD and ALDNC) exhibited significantly lower transient elastography values, than the ALDC patients (**Fig. 1E**). Moreover, incidence of steatosis was similar in NAFLD and ALDNC patients, while not a single case of steatosis was observed in the ALDC group (**Fig. 1F**). These effects are in-line with previously published results in similar patient cohorts.

### Surrogate cell death markers and TNF-alpha discriminate NAFLD and ALD with and without cirrhosis

**Figure 2:**
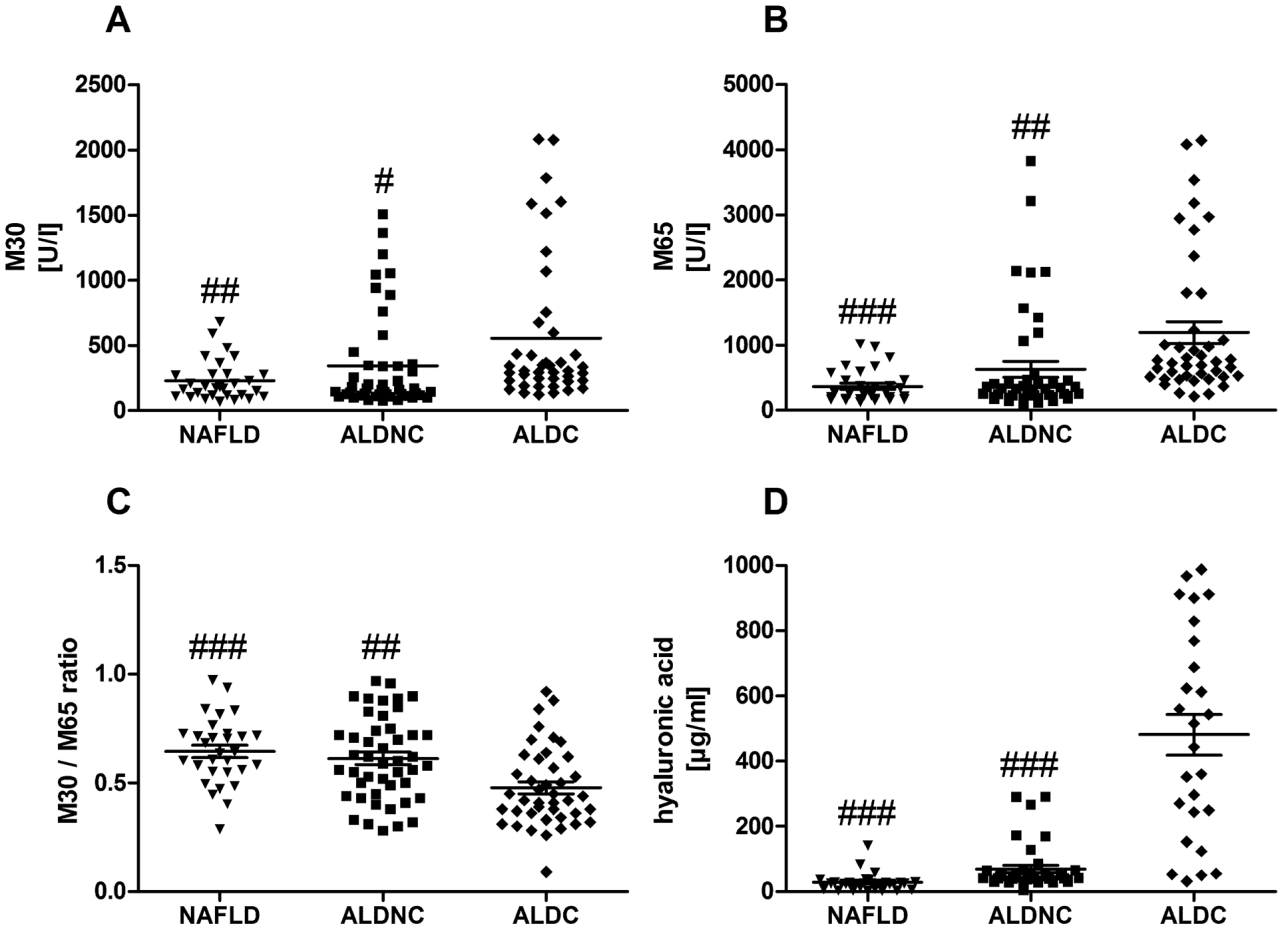
Elevation in serum cell death markers is specific for cirrhosis status but not for etiologies. Surrogate serum markers of apoptosis (M30, A) and general cell death (M65, B) were measured in NAFLD and ALD patients with (ALDC) or without (ALDNC) cirrhosis. Both markers were found elevated in all groups, with ALDC exhibiting significantly higher values than NAFLD or ALDNC patients. The ratio of M30/M65 (C) gives an estimate of the main cell death mode (predominantly apoptosis or necrosis). This ratio was significantly lower in ALDC compared to both non-cirrhotic groups, suggesting predominantly necrotic processes. Hyaluronic acid (D) as derivate marker for collagen production was significantly higher in ALDC patients than in the non-cirrhotic groups. #, ##, ### = *p*<0.05, 0.01 or 0.0001 *vs.* ALDC.

Cytokeratin 18 served as serum marker for apoptotic cell death (caspase cleaved epitope: M30) and overall cell death (total CK-18: M65). ALDC patients exhibited significantly higher M30 and M65 levels than NAFLD or ALDNC patients, respectively (**Fig. 2A,B**). Calculating the ratio of M30 to M65 gives a rough estimate of the predominant cell death mode (apoptotic *vs.* necrotic) [15]. In the presented patient groups, this ratio was highest in NAFLD and lowest in ALDC, suggesting a stronger contribution of necrotic cell death in cirrhotic ALD (**Fig. 2C**). The difference between ALDC and NAFLD as well as ALDNC was statistically significant. Hyaluronic acid serves as surrogate marker for fibrotic liver [16], [17]. As expected, highest hyaluronic acid serum concentrations were found in ALDC (**Fig. 2D**).

### Serum adiponectin reduction in NAFLD is contrasted by high elevation of adiponectin concentrations in ALD patients

**Figure 3:**
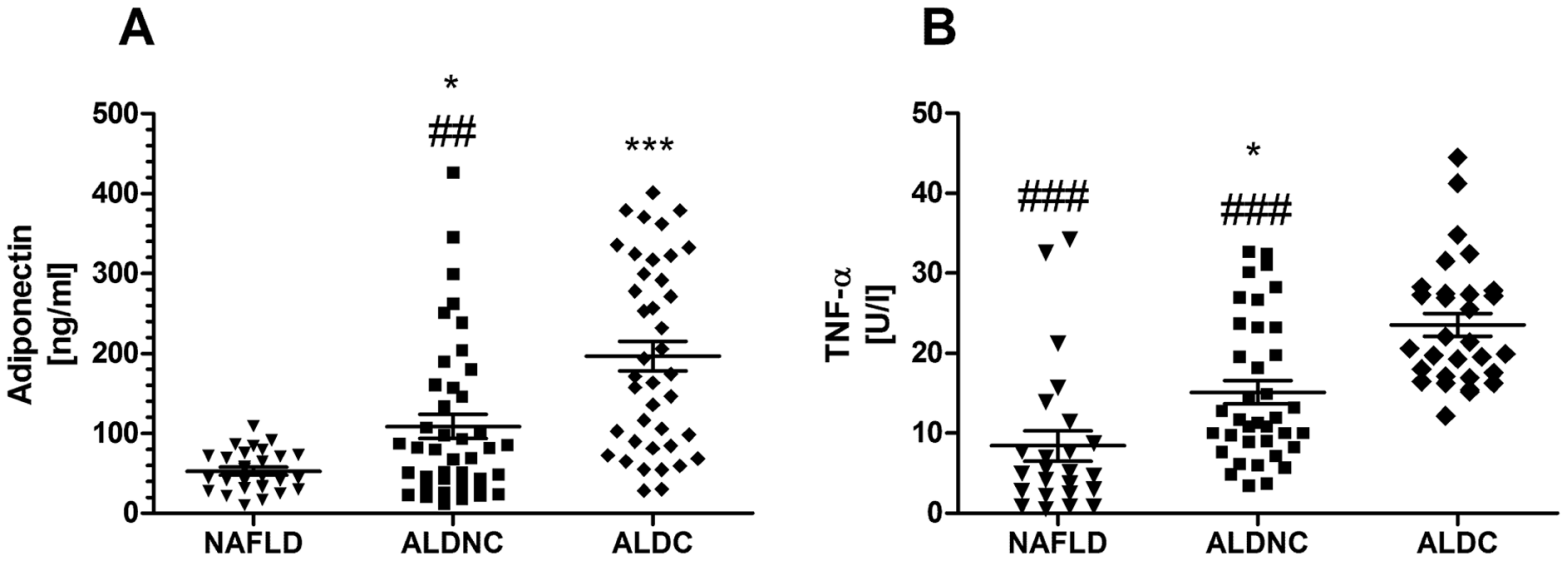
Adipocytokine profiles differ between NAFLD and ALD patients regardless of cirrhotic alterations. Adipokines are cytokines produced by the adipose tissue, which may affect other organ systems, including the liver. Adiponectin (A) is an anti-inflammatory and probably cell-protective adipokine, which is low in obese patients. In the NAFLD group (note: mean BMI 25.6) reduced adiponectin serum concentrations were found. Differences to both ALD groups were significant. While the ALDNC group exhibited values around normal ranges and above, the adiponectin levels in cirrhotic ALD were strongly increased and significantly different from the non-cirrhotic group. TNF-alpha (B) is a pleiotropic, generally pro-inflammatory cytokine. Surprisingly serum TNF-alpha was low in NAFLD, with significantly higher values in non-cirrhotic ALD. In ALDC patients a strong elevation of serum TNF-alpha was observed, which was significant compared to both non-cirrhotic groups. *, *** = *p*<0.05 or 0.0001 *vs.* NAFLD. ##, ### = *p*<0.01 or 0.0001 *vs.* ALDC.

The adipokine adiponectin is produced by “lean” adipocytes and is decreased in obese individuals [12], [13]. As prior findings of others and our own group would suggest, we found reduced adiponectin levels in NAFLD patients with “low” BMI (**Fig. 3A**). To the contrary, adiponectin in the serum of both ALD cohorts NC and in particular of ALDC patients were significantly increased. Concentrations above normal ranges (approx. 100 ng/ml) were observed. TNF-alpha is found in higher serum concentrations in obese and may contribute to a generally proinflammatory state in these individuals. In contrast to these previous findings, serum TNF-alpha was found in lowest concentrations in this cohort of NAFLD patients (near normal range), with higher amounts in ALDNC and highest levels in ALDC (**Fig. 3B**).

### Computational models can discern alcoholic and non-alcoholic fatty liver disease

**Figure 4:**
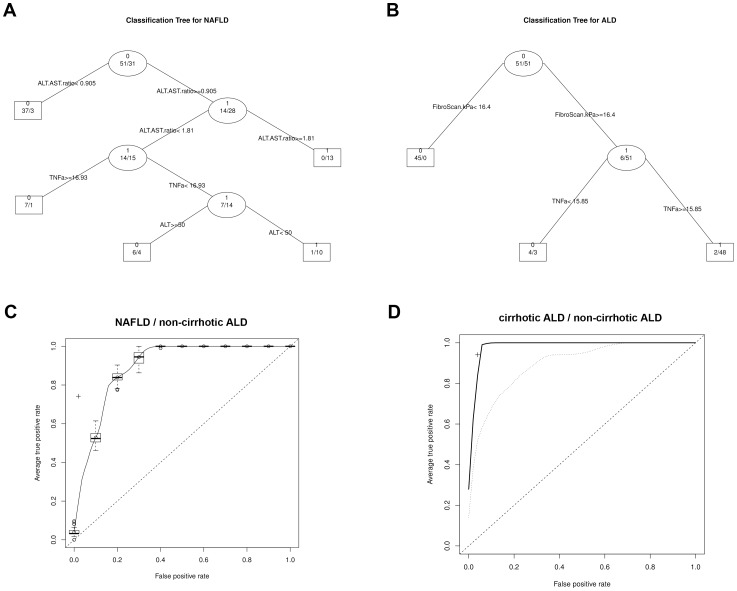
ROC curves for random forest group discrimination. Decision trees are shown for the classification of NAFLD *vs.* non-cirrhotic ALD (A) and cirrhotic *vs.* non-cirrhotic ALD (B), respectively. Move to the left branch when the stated condition is true, otherwise move to right branch. C: ROC curve for the RF (NAFLD *vs.* non-cirrhotic ALD); a + marks the performance of the corresponding DT; D: ROC curves for the RFs (cirrhotic *vs.* non-cirrhotic ALD), solid line: with transient elastography (FibroScan), dashed line: without transient elastography; a + marks the performance of the corresponding DT. The dotted line represents a classification by chance.

To calculate a predictive algorithm the above described parameters were introduced into four different machine learning approaches. The single DT was able to classify between ALDNC and NAFLD with a sensitivity of 74.19%, specificity of 98.04%, and an accuracy of 89.02% (the corresponding DT is shown in **Fig. 4A**). It was also possible to discern ALDC and ALDNC with a sensitivity of 94.12%, specificity of 96.08%, and an accuracy of 95.1% (**Fig. 4B**). ROC curves were plotted to assess sensitivity and specificity of the RFs (**Fig. 4C,D**). In the presented patient cohorts the RFs reached highly significant predictions with an AUC of 0.8932±0.0052 (*p*<0.0001 for NAFLD *vs.* ALDNC) and 0.9846±0.0018 (*p*<0.0001 for ALDC *vs.* ALDNC). When transient elastography measurements were excluded, to avoid confirmation bias, the AUC for ALDC *vs.* ALDNC reached 0.8971±0.0051 (*p*<0.0001). For comparison, the SVMs reached 0.9118±0.0056 (*p*<0.0001) for NAFLD *vs.* ALDNC and 0.9058±0.0035 (*p*<0.0001) for ALDC *vs.* ALDNC, respectively. The logistic regression performed slightly worse with 0.8893±0.0000 (*p*<0.0001; NAFLD *vs.* ALDNC) 0.8816±0.0000 (*p*<0.0001; ALDC *vs.* ALDNC).In addition to providing highly accurate models, RFs are able to estimate the importance of each variable to the classification process. Within each RF the most important parameters for discrimination of the classes (NAFLD *vs.* ALDNC and ALDNC *vs.* ALC, respectively) were calculated. The corresponding results are presented in **table 2**.

**Table 2:** Importance^1^ of entered parameters estimated by the RFs.

Parameter	Importance for NAFLD vs. ALDNC	Importance for ALDC vs. ALDNC	Importance for ALDC vs. ALDNC (transient elastography excluded)
Gender	0.57	0.20	0.49
Age	3.67	3.53	6.84
AST	1.65	2.63	4.84
ALT	5.15	1.18	3.00
ALT/AST ratio	10.41	1.39	3.68
M30	2.25	3.27	5.88
M65	2.65	6.21	11.96
TNF-alpha	5.6	5.52	7.65
Adiponectin	3.38	3.27	6.05
Transient elastography	2.66	23.25	-

1 Higher values imply greater importance for the decision.

## Discussion

Assessment of the cause for a metabolic liver disease remains one of the current clinical difficulties. In the presented patient cohorts, a possible mode of separation between alcoholic and non-alcoholic liver disease patients *via* serum derived measurements is suggested. Separation of these causes for metabolic liver injury is important not only for conservative treatment of patients, but also crucial for the decision making processes for liver transplantation and organ allocation. The long-standing observation that NAFLD and ALD differ in the ALT/AST ratio was confirmed in our patient collective; a high ratio indicates NAFLD, while a low ratio is associated with ALD. This work also identified two new markers which could help delineate between ALD and NAFLD. These markers are the adipokine adiponectin and the cytokine TNF-alpha. Especially low adiponectin, generally associated with obesity and thus NAFLD, may be a highly valuable marker due to its specific production site (adipose tissue) and the clear distinction between a very low concentration even in NAFLD with moderately high BMI, and common concentrations in ALD in a similar BMI range.

Another important aspect of the presented findings is the difference between ALD patients with a rather mild liver injury (ALDNC) and those with end-stage cirrhotic alterations, under similar habits of alcohol consumption. Somewhat expected were higher levels of surrogate markers for cell death and collagen production. Though, again adiponectin and TNF-alpha stood out as significantly different between ALD patients with and without cirrhosis. In particular, the strong elevation of anti-inflammatory adiponectin in ALDC patients suggests a disturbed metabolic regulation in this group. Not as surprising, but still notable, is a stronger elevation of TNF-alpha in the same group. Again, one has to keep in mind that groups did not differ in the amount of alcohol consumption. This finding could imply a possible functional involvement of adiponectin or its liver receptor ApoRII for progression of ALD to cirrhosis. Indeed, in hepatitis C virus-infected patients ApoRII expression correlates with serum adiponectin, steatosis, and liver fibrosis [18]. Increased adiponectin levels, without an actual protective effect might even indicate a crosstalk from liver to adipose tissue, initiating a compensatory mechanism [19]. Further studies are warranted to establish adiponectin as possible marker for monitoring of metabolic liver diseases. Furthermore, the current lack in mechanistical understanding of adiponectin signaling within the liver and the regulatory mechanisms in adipose tissue for adiponectin production should be targeted to evaluate this axis as drug target for ALD or NAFLD.

A crucial result of the presented work is the ability of a small set of non-invasive parameters to discern NAFLD and ALD, as shown by the calculated machine learning methods. One major advantage of the presented algorithms is the wide availability of the used parameters. Self reported consumption of alcohol is not always reliable to establish either NAFLD or ALD. From a clinical perspective it would be highly valuable to confirm or exclude ALD with high probability, without the need to rely on information given by the patient.

Similarly RFs and DTs were able to discriminate between ALDC and ALDNC with very high accuracy. This was mainly due to inclusion of transient elastography, which can detect cirrhosis reliably when ascites or other disturbing factors are absent [20], [21]. Unfortunately this simple and highly informative method is not widely available, as a special ultra-sound head is needed to perform transient elastography measurements on tissue. Moreover, during patient recruitment cirrhosis was assessed by conventional ultra-sonography and transient elastography was performed as additional parameter. Though, to avoid confirmation bias from two sonographic methods, a second model to discriminate ALDC and ALDNC was calculated without transient elastography, which again yielded significant results.

RFs and DTs offer the ability to assess importance of variables used for classification in a specific model. These importance values can be used to find a minimal set of variables for the classification, thus reducing the amount of parameters which need to be determined and thus cost of a possible clinical application. Furthermore, assessment of importance enables more insights into the classification process and might even suggest underlying biological interactions, identifying interesting targets for disease monitoring or therapy. This is a clear advantage of DTs and RFs compared to other machine learning techniques that are rather black boxes, such as SVMs. It is noteworthy that in this model serum parameters of cell death and cytokines were the most important parameters for decision making. A previous approach for non-invasive fibrosis assessment in NAFLD yielded similar results [22]. While the classic liver serum parameters are still important, as seen for discerning NAFLD and ALD, additional parameters as cell death markers, cytokines and adipokines should be collected routinely to monitor disease progression or for diagnostic purposes. Broad usage of those parameters may confirm current data in larger proportions of the general population.

Limitations of the current study are the unavailability of liver tissue biopsies from the majority of patients. This unfortunately not only restricts exact pathological assessment (steatosis as well as fibrosis stages) but also excludes studies on cellular or molecular processes. For example it would be highly interesting to investigate expression of PAI-1 in the liver, as an important candidate for alcohol mediated inflammatory damage and fibrogenesis [23], [24]. Differences between NAFLD and ALD or the different extent of damage in ALD might support the supposed functional involvement of PAI-1 in progression of ALD. Similarly interesting would be if expression of the adiponectin receptor ApoRII in the liver tissue correlates with severity of cirrhosis. Another limiting aspect is the relatively small number of NAFLD patients. This is partially due to the intention of comparing physiological similar patients with NAFLD and ALD. As the majority of definite NAFLD patients are obese, restriction to BMI of below 30 reduced the available number of patients. Finally, one limitation is represented by missing follow ups on the patients to assess development, progression or recession of the liver damage during disease course.

Taken together it could be shown that adipokines/cytokines may serve as markers for identification of NAFLD vs. ALD. This would enable clinicians to cross-check the information given by patients about their alcohol consumption with minor additional expenses but with high accuracy. In addition, severity of ALD may be non-invasively diagnosed *via* serum cytokine concentrations. Adiponectin or its receptors might even exhibit functional and thus therapeutic relevance in the progression of ALD to cirrhosis.

## References

[1] Bhala N, Jouness RIK, Bugianesi E (2013) Epidemiology and natural history of patients with NAFLD. Curr. Pharm. Des 19: 5169–5176. 23394091 10.2174/13816128113199990336

[2] Zakhari S, Li T-K (2007) Determinants of alcohol use and abuse: Impact of quantity and frequency patterns on liver disease. Hepatology 46: 2032–2039. 18046720 10.1002/hep.22010

[3] Erim Y, Beckmann M, Tagay S, Beckebaum S, Gerken G, et al (2006) [Stabilisation of abstinence by means of psychoeducation for patients with alcoholic liver disease awaiting liver transplantation]. Z Psychosom Med Psychother 52: 341–357. 17156604 10.13109/zptm.2006.52.4.341

[4] Cello JP, Rogers SJ (2013) Morbid obesity-the new pandemic: medical and surgical management, and implications for the practicing gastroenterologist. Clin Transl Gastroenterol 4: e35. 23739585 10.1038/ctg.2013.6 PMC3696938

[5] Ertle J, Dechêne A, Sowa J-P, Penndorf V, Herzer K, et al (2011) Non-alcoholic fatty liver disease progresses to hepatocellular carcinoma in the absence of apparent cirrhosis. Int. J. Cancer 128: 2436–2443. 21128245 10.1002/ijc.25797

[6] Dunn W, Sanyal AJ, Brunt EM, Unalp-Arida A, Donohue M, et al (2012) Modest alcohol consumption is associated with decreased prevalence of steatohepatitis in patients with non-alcoholic fatty liver disease (NAFLD). J. Hepatol 57: 384–391. 22521357 10.1016/j.jhep.2012.03.024 PMC3399018

[7] Ruhl CE, Everhart JE (2005) Joint effects of body weight and alcohol on elevated serum alanine aminotransferase in the United States population. Clin. Gastroenterol. Hepatol 3: 1260–1268. 16361053 10.1016/s1542-3565(05)00743-3

[8] Erim Y, Böttcher M, Dahmen U, Beck O, Broelsch CE, et al (2007) Urinary ethyl glucuronide testing detects alcohol consumption in alcoholic liver disease patients awaiting liver transplantation. Liver Transpl 13: 757–761. 17457868 10.1002/lt.21163

[9] Meyer D, Leisch F, Hornik K (2003) The support vector machine under test. Neurocomputing 55: 169–186.

[10] Breiman L (2001) Random Forests. Machine Learning 45: 5–32.

[11] Cawley GC, Talbot NLC (2004) Fast exact leave-one-out cross-validation of sparse least-squares support vector machines. Neural Netw 17: 1467–1475. 15541948 10.1016/j.neunet.2004.07.002

[12] Fawcett T (2006) An introduction to ROC analysis. Pattern Recognit. Lett 27: 861–874.

[13] Sing T, Sander O, Beerenwinkel N, Lengauer T (2005) ROCR: visualizing classifier performance in R. Bioinformatics 21: 3940–3941. 16096348 10.1093/bioinformatics/bti623

[14] Cohen JA, Kaplan MM (1979) The SGOT/SGPT ratio–an indicator of alcoholic liver disease. Dig. Dis. Sci 24: 835–838. 520102 10.1007/BF01324898

[15] Kramer G, Erdal H, Mertens HJMM, Nap M, Mauermann J, et al (2004) Differentiation between cell death modes using measurements of different soluble forms of extracellular cytokeratin 18. Cancer Res 64: 1751–1756. 14996736 10.1158/0008-5472.can-03-2455

[16] Frébourg T, Delpech B, Bercoff E, Senant J, Bertrand P, et al (1986) Serum hyaluronate in liver diseases: study by enzymoimmunological assay. Hepatology 6: 392–395. 3710427 10.1002/hep.1840060310

[17] Guéchot J, Poupon RE, Poupon R (1995) Serum hyaluronan as a marker of liver fibrosis. J. Hepatol 22: 103–106. 7665843

[18] Wedemeyer I, Bechmann LP, Odenthal M, Jochum C, Marquitan G, et al (2009) Adiponectin inhibits steatotic CD95/Fas up-regulation by hepatocytes: therapeutic implications for hepatitis C. J. Hepatol 50: 140–149. 19019483 10.1016/j.jhep.2008.08.023

[19] Bechmann LP, Hannivoort RA, Gerken G, Hotamisligil GS, Trauner M, et al (2012) The interaction of hepatic lipid and glucose metabolism in liver diseases. J. Hepatol 56: 952–964. 22173168 10.1016/j.jhep.2011.08.025

[20] Sagir A, Erhardt A, Schmitt M, Häussinger D (2008) Transient elastography is unreliable for detection of cirrhosis in patients with acute liver damage. Hepatology 47: 592–595. 18098325 10.1002/hep.22056

[21] Vizzutti F, Arena U, Nobili V, Tarquini R, Trappoliere M, et al (2009) Non-invasive assessment of fibrosis in non-alcoholic fatty liver disease. Ann Hepatol 8: 89–94. 19502649

[22] Sowa J-P, Heider D, Bechmann LP, Gerken G, Hoffmann D, et al (2013) Novel Algorithm for Non-Invasive Assessment of Fibrosis in NAFLD. PLoS ONE 8: e62439. 23638085 10.1371/journal.pone.0062439 PMC3640062

[23] Beier JI, Arteel GE (2012) Alcoholic liver disease and the potential role of plasminogen activator inhibitor-1 and fibrin metabolism. Exp. Biol. Med. (Maywood) 237: 1–9. 22238286 10.1258/ebm.2011.011255 PMC5047512

[24] Verrijken A, Francque S, Mertens I, Prawitt J, Caron S, et al (2014) Prothrombotic factors in histologically proven nonalcoholic fatty liver disease and nonalcoholic steatohepatitis. Hepatology 59: 121–129. 24375485 10.1002/hep.26510

